# Protective immune response induced by cationic liposomes bearing soluble antigens improves the survival of BALB/c mice against *Toxoplasma gondii* RH strain

**DOI:** 10.22038/ijbms.2024.82123.17770

**Published:** 2025

**Authors:** Hadi Mirahmadi, Ahmad Mehravaran, Mahdi Kavand, Ali Reza Salimi Khorashad, Nasrin Rezaee

**Affiliations:** 1 Department of Parasitology and Mycology, Faculty of Medicine, Zahedan University of Medical Sciences, Zahedan, Iran; 2 Clinical Immunology Research Center, Zahedan University of Medical Sciences, Zahedan, Iran; 3 Infectious Diseases and Tropical Medicine Research Center, Research Institute of Cellular and Molecular Science in Infectious Diseases, Zahedan University of Medical Sciences, Zahedan, Iran; 4 Health and Treatment Network of Mehran City, Ilam University of Medical Sciences, Ilam, Iran

**Keywords:** Adjuvant, Cationic liposome, Immune response, Survival rate, Toxoplasma gondii

## Abstract

**Objective(s):**

An ideal strategy to control acute or chronic toxoplasmosis can be the development and production of an effective vaccine. Liposomes as immunoadjuvants may be utilized to boost immune reactions for various antigens.

**Materials and Methods:**

In this study, we encapsulated soluble Toxoplasma antigen (SA) in 1, 2-Dioleoyl-3-trimethylammonium propane (DOTAP) liposomes to assess the elicited immunological response. BALB/C mice received three intramuscular injections of various formulations separated by two weeks. The kind of immune reaction that was created, the degree of protection, the percentage of BALB/c mice that survived the *Toxoplasma gondii* challenge, the immune reaction assessment with cytokine synthesis (IFN-γ, IL-4), and the titration of IgG isotypes were all evaluated.

**Results:**

Compared to other groups, the liposome DOTAP + imiquimod + SA-immunized mice showed a significantly lower death rate (*P*<0.01). Liposome DOTAP + Imiquimod + SA had higher IgG2a and IFN-γ secretion levels than the control group (*P*<0.001 and *P*<0.0001, respectively).

**Conclusion:**

According to the study’s findings, the liposome DOTAP + imiquimod + SA formulation generates a cellular immunological response, making it resistant to the *T. gondii *challenge.

## Introduction

*Toxoplasma gondii*, an obligatory intracellular parasite, belongs to the phylum Apicomplexa and is found worldwide. Humans and other warm-blooded animals are frequently infected. Although infections can occasionally result in malignant illness, they are mostly asymptomatic. (1). The *T. gondii* infection may be lethal in immunocompromised patients, such as AIDS or solid organ transplantation generally (2, 3). In pregnant women, an acute and primary infection may also cause disabling and severe disease in the growing fetus (4). Humans usually become infected through oral intake of viable tissue cysts in undercooked meat or water and food intake contaminated by oocysts from feline feces (5). 

Developing a vaccine against toxoplasmosis is a serious concern to prevent the disease’s harmful effects (6). The current strategy to create a long-lasting, effective vaccine is to use parasitic antigens, such as surface tachyzoites and bradyzoites proteins. However, these antigens do not have enough immune function alone and need proper adjuvants for better performance. Immune stimulation, presentation, uptake, and efficient and safe adjuvant co-administration are necessary to enhance the persistence of antigens. A desirable approach to an effective vaccine plan is integrating delivery systems and immunopotentiating adjuvants (7, 8). Dendritic cells (DCs), neutrophils, and monocytes are involved in the infection site, which is challenged with *T. gondii *(9). Numerous nanocarriers, including liposomes, polymerases, micelles, and archaeosomes thus far, have been utilized to carry protein antigens to antigen-presenting cells (APCs) (10, 11). Bilayer vesicles containing aqueous molecules and liposomes have been used as delivery methods for medicines, proteins, and peptides. Liposomes might also improve immune responses to various antigens by acting as immunoadjuvants. Antigens may be connected to these structures in numerous ways, such as antigen encapsulation inside the liposome’s lipophilic center, lipid bilayer, antigen’s transmembrane regions, and surface adsorption (12, 13). Cationic liposomes as delivery vehicles are appealing and promising due to their clinical safety, storage properties, low immune stimulation, and simple preparation. The latest investigations have also revealed that Cationic liposomes containing DOTAP serve as vaccine delivery vehicles and enhance the suboptimal immunogenic profiles of peptide/protein antigens while also inducing Th1 and cytotoxic T lymphocyte (CTL) responses. (14).

The ability of some substances to enhance the efficacy of the vaccine was reported first with aluminum salts, called adjuvants. These compounds can increase immunogenicity and vaccination efficacy through antigen transport, stabilization, and enhanced innate immune activation. Adjuvant interacts with different receptors, including TLRs (Toll-like receptors), and activates the immune response over multiple mechanisms based on their particular features (15). TLRs 3, 7, 8, and 9, from among the 11 mammalian TLRs, are found in the cell endosomes; they identify the intracellular DNA and RNA pathogens (16). Imiquimod (R837) adjuvant is a synthetic agent with immune response modifying activity. Moreover, once administered with the antigen, the imiquimod occurrence in the vaccine design may improve the immune reaction. Imiquimod not only does not have properties of immunomodulatory activity but also imposes certain impacts on different engaged cells in the system of immune and prompts some cytokines release, such as the tumor necrosis factor (TNF)-α, interferon (IFN-γ), interleukin (IL)-1b, IL-8, and IL-6 (17). This investigation aimed to determine if DOTAP liposomes were immunogenic with soluble Toxoplasma antigen (STA=SA) and imiquimod adjuvant against *T. gondii* challenge in BALB/c mice. More precisely, we are looking for whether cationic liposomes with imiquimod adjuvant can increase the survival rate of BALA/c mice against *T. gondii *infection.

## Materials and Methods

### Animals and ethical statement

Female BALB/c mice, aged 6–8 weeks, utilized in this work were acquired from the Laboratory of Animal Research Center of Zahedan University of Medical Sciences in Zahedan, Iran. The mice were held pathogen-free in an animal care kit. The animals were housed at 21 °C with unrestricted access to food and water in a colony room with a 12/12 light/dark cycle. The procedure was approved by the Institutional Ethical Committee and Research Advisory Committee of Zahedan University of Medical Sciences following the Guidelines of Specific National Ethical for Biomedical Research published by the Research and Technology Deputy of the Ministry of Health and Medicinal Education (MOHME) in Iran (Education Office; dated March 31, 2010; proposal code, 88527).

### Imiquimod, parasites, and soluble antigen (SA)

Mazandaran University of Medical Sciences’ Toxoplasmosis Research Center in Sari, Iran, provided the strain of *T. gondii* (RH) used in this investigation. We purchased imiquimod adjuvant (R837) from the Invivogen Company (USA). A modified process was put into place for SA preparation. In conclusion, three rounds of washing with HEPES buffer (10 mM + 10% sucrose, pH 7.4) were performed on the fresh and active tachyzoites that proliferated in BALB/c mice injected intraperitoneally. After that, these tachyzoites were lysed in an ice bath by combining probe sonication with a freeze-thaw cycle. After centrifuging the resultant lysate, the supernatant was dialyzed against HEPES buffer, sterilized using a 0.22 μm filter, and kept at -70 °C for storage. (18). The SA protein concentration (1mg/ml) is shown using a protein assay kit (BCA) (Thermo Scientific, USA) (19).

### Preparation and characterization of liposomes

The lipid film method was used to create liposomes. A sterile tube was filled with chloroform to dissolve the lipid phase, which included cholesterol (10 mM; Avanti polar lipids, USA) and DOTAP (20 mM; Avanti polar lipids, USA) in a 1:2 molar ratio. Following the removal of the solvent by rotary evaporation (Hettich, Germany), a thin lipid coating was deposited on the tube wall. The solvent was eliminated from the lipid layer by overnight freeze-drying (TAITEC, Japan) for complete solvent evaporation. Then SA (1 mg/ml) was added to a sterile solution (HEPES buffer 10 mM pH 7.4) to dilute and disperse the lipid film. A bath sonicator (Bandelin, Germany) was used to convert multilamellar vesicles (MLVs) into unilamellar vesicles for five minutes at 45 °C. The liposome was expelled thirteen times through polycarbonate membranes with diameters of 200 and 400 nm (Avestin, Canada). A Dynamic Light Scattering Instrument (Nano-ZS, Malvern, UK) was employed to ascertain the produced liposomes’ particle size and zeta potential. The polydispersity index (PDI) and the mean ± standard deviation (n=3) reflected particle sizes. On a similar machine, through the zeta potential mode, the means±zeta deviation (n=3) was used to determine the zeta potentials. The liposome size and form were investigated using transmission electron microscopy (TEM, Jeol JEM-1220, JEOL, Japan). The dialysis method was used 24 hr to purify the liposomes and remove the antigens (SA) that did not enter the liposomes. PBS buffer with pH 7.2 was used for size and zeta analysis (20).

### Characterization of the provided formulations

The BCA protein assay kit was used to define the antigen and determine the liposome’s SA concentration (Thermo Scientific, Waltham, USA). The SA present in the liposomal SA (Lip-SA) was qualitatively identified using analytical SDS-PAGE. Stacking and running gel with a thickness of 1 mm (12.5% and 4.78% w/v acrylamide, respectively) were part of the discontinuous system. The electrophoresis buffer consisted of 0.1% SDS at pH 8.3, 25 mM Tris, and 192 mM glycine. For 45 min, electrophoresis was conducted at a constant voltage of 140 V. For each well, the same quantity of SA (2.5 or 5µg) was added for each formulation. Silver staining was applied to the gels to identify proteins following electrophoresis (21).

### Immunization and challenge

The primary objective of the research, as specified in the introduction, was to investigate if DOTAP-based designs of liposome with SA and Imiquimod adjuvant combination may activate the immune system. Various vaccine formulations, therefore, were picked up for that reason. Fifty BALB/c mice (6–8 weeks old) were randomly assigned to five groups (10 mice in each group). Each group received intramuscular immunizations in both quadriceps (right and left) on three occasions, spaced three weeks apart, with one of the following formulations: SA with DOTAP liposome, SA with PBS (Phosphate-buffered saline) buffer, SA with Imiquimod, and SA with liposome DOTAP and Imiquimod. A syringe carrying 100 μl of the formulation (sterile PBS containing 50 μg of antigen) was used for each injection. Before injecting 50 μl into each muscle, the injection site was cleaned with alcohol. Three rounds of vaccinations were administered in the prescribed order on days 0, 21, and 42. Two weeks after the final booster, the mice received 1 x 10⁴ tachyzoites of the *T. gondii* RH strain intraperitoneally, and the length of time they survived was noted daily (22).

### Antibody type assay

An indirect enzyme-linked immunosorbent test (ELISA) was used to determine the amounts of serum IgG specific to antigens. Blood drawn from the venous plexus in the mice’s tails between weeks six and nine following inoculation was centrifuged to separate mouse sera from each group, and then the sera were stored at -20 °C. ELISA was then used to measure the amounts of IgG, IgG1, and IgG2a antibodies specific to *T. gondii*. In brief, After coating 96-well microtiter plates with 20 μg of SA in 1 ml PBS and incubating them overnight at 4 °C, they were blocked for two hours at room temperature using 2% BSA in PBS. Mouse sera were incubated for two hours at 37 °C after being diluted 1:100 in 1% BSA-PBS. After three PBST washes, the plates were incubated for two hours at 37 °C with goat anti-mouse IgG, IgG1, and IgG2a antibodies (Invitrogen Inc., USA) diluted 1:5000 and conjugated with horseradish peroxidase. Adding a substrate solution (pH 4.0) containing 1.05% citrate substrate buffer, 1.5% ABTS, and 0.03% H_2_O₂ allowed for the visualization of the immunological complexes. A microplate reader (Stat Fax 2100, Awareness, Palm City, FL, USA) was used to measure the optical density at 450 nm after stopping the reaction with 1M H₂SO₄. All samples were tested in triplicate (23).

### Cytokine assay

In order to analyze the cytokine production level, three mice from each group were slaughtered at week nine following inoculation, and their spleens were aseptically removed. Ficoll-Hypaque density centrifugation was used to separate mononuclear cells from the spleen cell solution. After being cleaned and resuspended in RPMI 1640-FCS, the cells were seeded in 96-well flat-bottom plates (Nunc, Denmark) at 2 × 106/ml density. *T. gondii* lysate antigen (TLA) (10 μg/ml), Con A (2.5 μg/ml), or medium alone were used to stimulate the cells *in vitro*. They were then incubated for 72 hr at 37 °C with 5% CO_2_. Following the manufacturer’s instructions, the culture supernatants were collected, and ELISA kits (eBiosciences, San Diego, CA, USA) were used to measure the amounts of IL-4 and IFN-γ. Assays were carried out in three copies.

### Statistical analysis

In GraphPad Prism software (Version 9), the statistical difference in groups was documented and examined. The importance of the variants between different groups was evaluated through a one-way ANOVA statistical analysis. In order to assess the average in various mice groups, multiple comparisons of Tukey–Kramer were performed as a post-test. Statistically, *P*<0.05 appeared to be meaningful.

## Results

### Liposome characterization

**Figure 1:**
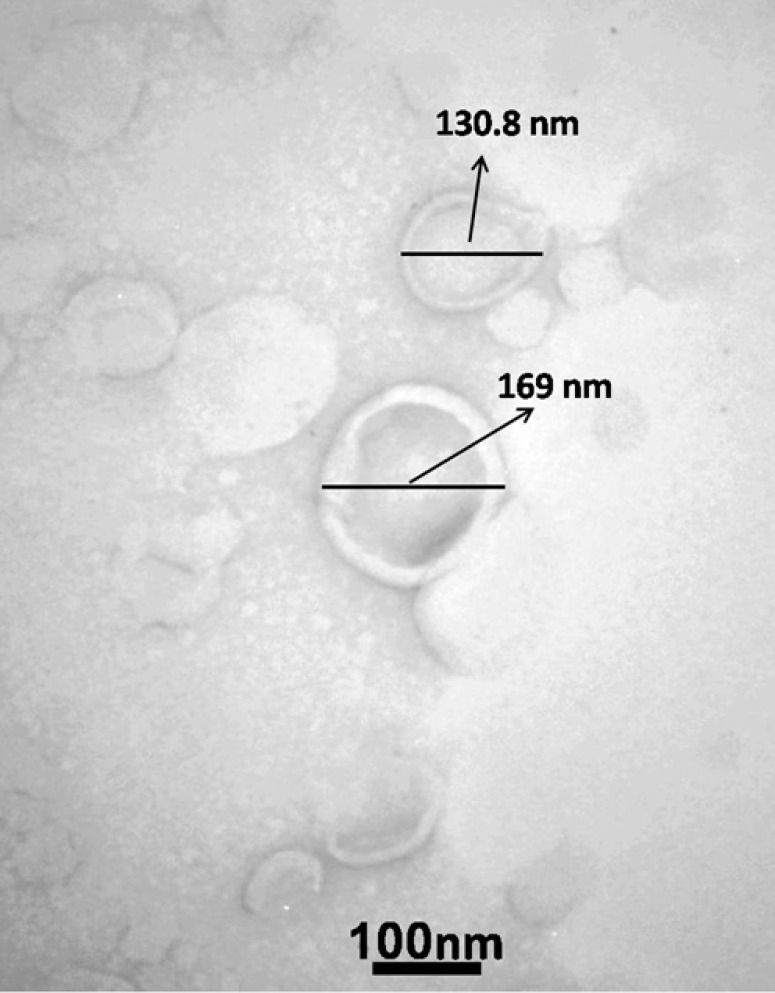
Detection of liposomal formulations by transmission electron microscopy (TEM)

The TEM analysis of the provided liposome+SA is illustrated in Figure 1. The single (un-agglomerated) spherical liposome nanoparticles were validated by TEM analysis. The average diameter values of the three liposome compositions were 217±15 nm, Intensity Mean: 232 nm, Volume Mean: 234.4 nm, and Number Mean: 190 nm. The zeta potentials of empty liposomes were 23.5±2 mV, and liposome+SA was 4.5±3. The antigen SA has a negative charge, and when it enters the liposome, it reduces the charge of the liposome to the negative side. The SA entrapment in liposomes was calculated at 57±14% (n = 3) (Table 1).

**Figure 2:**
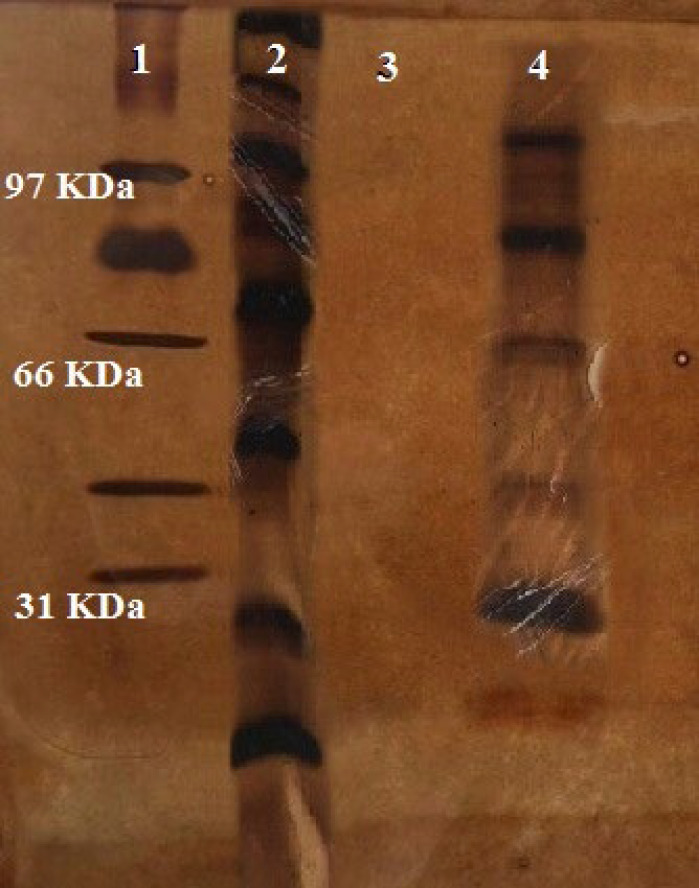
SA and liposomal SA SDS-PAGE analysis

Prior to injection, the compounds’ SA concentration was set at 50 μg/50 μl. SDS-PAGE electrophoresis was used to characterize SA and liposomal SA (Figure 2). The SDS-PAGE examination of the soluble antigen showed many protein bands with different ranges. Nearly all of the bands in the liposome SA analysis resembled the free SA, suggesting that SA proteins were trapped in the structure during liposome production.

### Antibody assay

**Figure 3:**
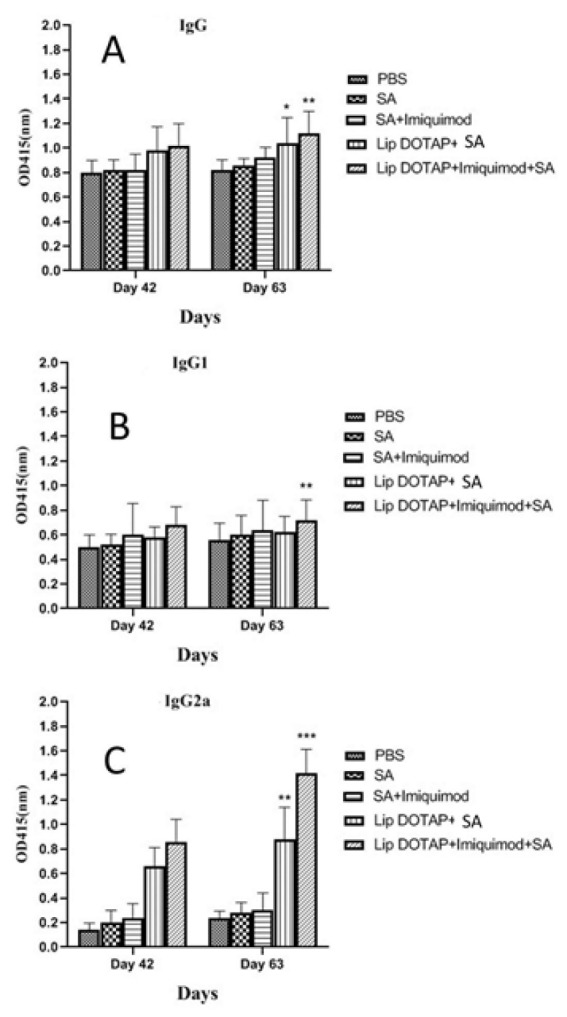
IgG (A), IgG1 (B), and IgG2a (C) anti-SA antibody levels in pooled sera of BALB/c mice inoculated intramuscularly (IM) with various formulations, including SA, SA + Imiquimod, liposome DOTAP + SA, liposome DOTAP + imiquimod + SA, or buffer alone

On days 42 and 63 after the initiation, the serum levels of anti-SA specific IgG1, IgG2a, and total IgG antibodies were measured in BALB/c mice to determine the sort of immunological response produced (Figure 3 A-C). When comparing the sera of the inoculated mice with different designs to the controls receiving PBS buffer alone on day 63 following the start of inoculation, a significant difference in the amounts of IgG1, IgG2a, and IgG Abs was seen; however, there was insignificant difference in the Abs levels on day 42 (Figure 3A-C). IgG2a levels in the serum of mice injected with liposome DOTAP + SA and liposome DOTAP + Imiquimod + SA were remarkably (*P*<0.01) and (*P*<0.001) higher on day 63 than on day 42 (Figure 3C). Additionally, on day 63 following inoculation, the rate of IgG1 was significantly different in the liposome DOTAP + Imiquimod + SA group compared to all vaccinated animals (Figure 3B).

### Cytokine assay

**Figure 4:**
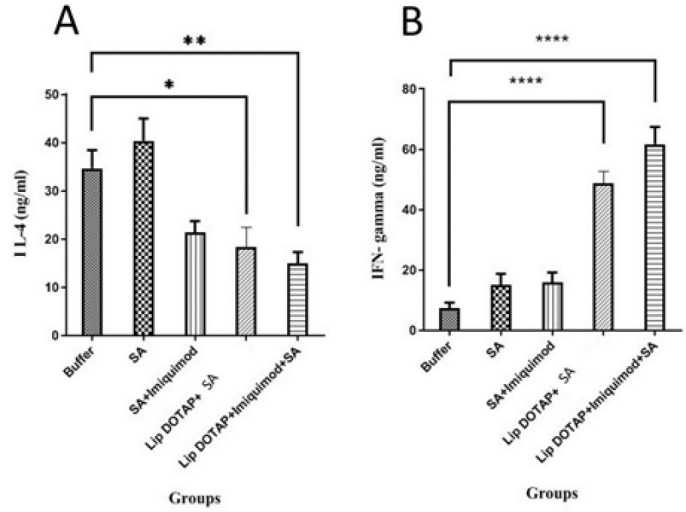
Assessment of the cytokine levels in challenged immunized mice

Different liposomal IFN-γ and IL-4 generation constructs were activated and computed via the ELISA technique to evaluate how well components trigger the cellular immune response (Figure 4A-B). The findings indicated that splenocytes detached from the immunized mice group, liposome DOTAP + Imiquimod + SA secreted meaningfully greater IFN-γ amounts + SA and liposome DOTAP in comparison to the immunized mice with the buffer (*P*<0.0001) (Figure 4B). However, the IL-4 volumes in the mice vaccinated by liposome DOTAP + SA and liposome DOTAP + Imiquimod + SA were considerably lower (*P*<0.05 and *P*<0.01), respectively, than in the mice group that received the buffer. Nevertheless, IL-4 production was detectable for all mouse groups (Figure 4A).

### Challenge results

**Figure 5:**
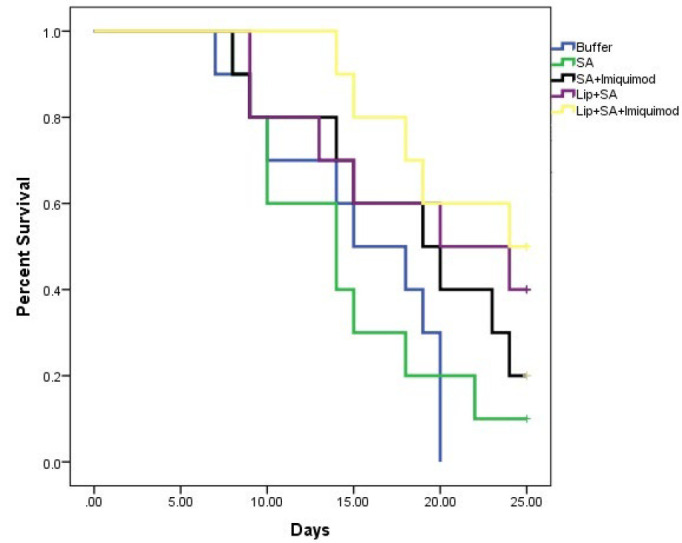
Survival rates of BALB/c mice immunized against *Toxoplasma gondii* using various formulations

The percent survival results with GraphPad Prism software showed that mortality in the study BALB/c mice started from day 10 and continued until day 25 (Figure 5). Compared to the mice vaccinated with liposome DOTAP + SA, liposome DOTAP + Imiquimod + SA, and SA + Imiquimod following the challenge, the survival percentage dropped sharply in the SA or buffer immunized mice. The mice injected with liposome DOTAP + SA + Imiquimod exhibited a considerably (*P*<0.01) higher survival rate than the buffer group on day 25 after the challenge.

## Discussion

Succeeding to obtain an effective vaccine formulation with long-term effects against *T. gondii* in the intermediate host, especially in humans, is an important and difficult challenge that is caused by the existence of ambiguities in the parasite pathogenesis and complexities of the immune response required for defensive purposes. Creating a vaccination that effectively prevents toxoplasmosis is challenging for several reasons. We address the three main reasons for the difficulties in developing a vaccine against Toxoplasma. The lack of a consistent vaccination schedule regarding the infectious dose or strain is the main driver behind developing a vaccine against *T. gondii*. For example, some laboratory strains, such as RH, do not match the native strains in terms of performance (24). The parasite’s multistage life cycle is the second significant factor; several studies have demonstrated that the immune response to one life cycle stage is ineffective against subsequent stages (25). The ability of the parasite to evade detection and modify the host immune system’s signaling pathways is the third essential component. For example, one of the dense granule proteins, TgIST, can suppress STAT1-dependent proinflammatory gene expression and IFN-γ production (25, 26). For the reasons stated, the most effective formulation seems to be a mix of multistage antigen vaccines or the development of fusion proteins or crude antigens that incorporate them.

Therefore, one crucial and significant stage in developing an effective vaccine to combat toxoplasmosis is choosing an appropriate delivery mechanism to elicit an effective immune response against it. Usual prophylactic vaccine approaches for intracellular pathogens, including Toxoplasma*, *suggest improving the innate immunity of the host and considering the adaptive reaction and pathogen by the vaccine. The development of protective immunity through vaccines relies on its ability to extract a suitable immune reaction to monitor or eliminate the pathogen. Most research concentrated on finding a proper vaccine for *T. gondii* protection by CD8+ subset of DCs creates IL-12 (27). Neutrophils, monocytes, and dendritic cells are recruited to the site of infection on challenge with *T. gondii*; thus, all contribute to resistance to this organism. On the other hand, questions have been raised about their unique role in infection control (28). The capability to diagnose pathogens and create the cytokine IL-12 is one of the most central tasks of the innate immune reaction to *T. gondii* that motivates natural killer and T cells to generate the cytokine interferon-gamma (29). The primary modulator of *T. gondii* resistance is IFN-γ, which activates several intracellular processes to eradicate the parasite and stop its reproduction. Infection characteristic by various intracellular pathogens is the Th1 immune reaction well-defined through the IFN-γ and IL-12 production. Deficient in either IFN-γ or IL-12, mice infected with *T. gondii* develop severe and malignant disease, demonstrating an inability to manage the parasite burden similar to infections by other intracellular pathogens. (30). On the other hand, Cells Th2 produce interleukin-10 (IL-10), interleukin-5 (IL-5), and interleukin-4 (IL-4), strengthening the humoral immunity responses and reducing cellular immunity (31). Liposomal nanoparticles, protein antigens, and adjuvant molecules may be useful compounds in creating protective immunity by T cells. TLR agonists combined with liposomes can simultaneously affect antigen presentation and pattern recognition receptor (PRR) pathways and effectively improve T cell function. (32, 33).

The size of liposomes has a major impact on the kind of immune response produced, which is essential to consider when developing an effective liposomal vaccine delivery method for intracellular infections, including leishmania, TB, and toxoplasmosis. The results of previous studies show that liposomes as tiny as 100 nm stimulate the humoral immune response, whereas liposomes bigger than 100 nm in mean size encourage the CMI response. So, we used liposomes larger than 200 nm. Our results are generally similar to other research on intracellular parasites (34). Current research uses a highly immuno-stimulating adjuvant compound (cationic and Imiquimod liposomes) to establish soluble Toxoplasma antigens prepared and used. The immunity responses and protection level were examined in the mice model infected with toxoplasmosis. Factors like the sera’s antibody level in the infected mice were assessed to investigate the protection rate. Our results revealed that SA + liposome DOTAP + Imiquimod could improve the antibody reactions against the infection of *T. gondii*. The evaluation of IgG1 and IgG2a antigen isotypes was respectively taken into consideration as the pointer of Th2 and Th1 immunity reactions.

The examination of the tests in the ongoing study reveals the greatest quantity of IgG2 and IFN-γ, while the liposome DOTAP + SA + imiquimod group exhibits the lowest levels of IL-4. This group had the highest survival rate of mice compared to other groups. These results show that the cellular immune response is more than the humoral immune response. Th1 immunity response could be generated by Liposome DOTAP + SA + imiquimod strategy to protect the mice facing toxoplasmosis and result in long-time safety encountering cell infection. RH strain of *T. gondii* is more pathogenic and virulent than other strains due to the production of some invasive proteins and enzymes from the apical complex in the microorganism. One of the reasons for using the RH strain of Toxoplasma vaccination studies on mice is the high power of this parasite virulence, which facilitates follow-up of changes in cytokine production and immune pathways in mice over a shorter period. This strain can quickly kill some intermediate hosts, including Syrian mice, BALB/C, or Golden Hamster. However, the same strain (RH) can become a tissue cyst in intermediate hosts such as rats or rabbits and survive long without destroying its host. In other words, the virulence and pathogenicity of a microorganism are closely related to its host physiology (35). 

There is a question about whether the amount of IgG1 was reported to be high in the liposome DOTAP + SA + imiquimod group and how this affects the survival rate of the mice against *T. gondii*. This can be explained by several factors related to the immune response dynamics following vaccination. 

### Mixed Th1/Th2 response

The increased levels of both IgG1 and IgG2a suggest a mixed Th1/Th2 immune response. While IgG1 is typically associated with Th2 responses, the presence of IgG2a indicates a robust Th1 response. The predominance of IFN-γ, a hallmark of Th1 immunity, can stimulate B cells to produce IgG2a while still allowing for some IgG1 production due to the overall mixed response (36). 

### Regulatory mechanisms

The immune system employs regulatory mechanisms that can balance different types of responses. The presence of certain cytokines can modulate B cell activity and class switching, allowing for the simultaneous production of different antibody classes even under conditions favoring Th1 responses. The observed increase in serum levels of IgG1 alongside elevated IgG2a and IFN-γ, along with decreased IL-4, reflects a complex interplay between Th1 and Th2 immune responses following Toxoplasma vaccination. This mixed response can be attributed to the specific vaccine formulation, the cytokine milieu induced by vaccination, and the inherent regulatory mechanisms within the immune system that allow for diverse antibody production despite a predominant Th1 enviro nment (37).

The findings indicated Imiquimod-induced Th1-based reactions. Also, it was discovered that the BALB/c mice’s immune protection responses to the *T. gondii* infection challenge were triggered by the imiquimod and Toxoplasma antigens. The imiquimod effects may generally increase if a vaccine transfer technology, such as liposomes, is used for protein-based immunization; nevertheless, the cationic liposomal delivery strengthened the immunogenicity of the purified antigen. (35). Our previous study (38) demonstrated that a formulation of the vaccine using DSPC liposomes as carriers for soluble antigens of the Toxoplasma parasite, combined with Imiquimod adjuvants, elicited the highest cellular immunity in comparison to soluble antigens alone or the use of Imiquimod and antigen adjuvants without liposomal carriers. In the study of Abdollahi *et al.*, it was found that mannose-modified nanoliposome of E/S antigens reduced the parasitic burden and increased the survival time in BALB/c mice also induced a more powerful immune response against *T. gondii* when compared with excreted/secreted antigens alone. The results of the above two studies show that using cationic liposomes and soluble antigens increases the survival time and cellular immune response. These results are similar to our current research (39). 

Vaccine delivery systems, such as liposomes, improve the associated antigen uptake into APCs. Liposome acts as an adjuvant and stimulates immune responses by various antigens. Cationic liposomes protect easily-altered antigens and facing lysosomal degeneration; thus, they benefit from the electrostatic contact with adverse charges of cells. This formulation’s ability to act as a suitable antigen delivery technique to trigger responses of T-cells is shown by DOTAP-bearing cationic liposomes (40). Imiquimod is very important in selecting effective immunomodulators because it protects experimental models of toxoplasmosis. Imiquimod’s immunogenicity and security can activate APCs and extract cytokines, including IL-4, IL-12, and IFN-γ, through co-stimulatory molecule maturation, crucial for T and B cell maturation and activation (27). The immunomodulation effect on different cells triggers Imiquimod; these are efficient in the immune functions in that way induce secretion of cytokines, including the alpha factor of TNF-α, IFN-γ, IL-6, IL-8, as well as IL-1beta (41). The primary target cells of Imiquimod are macrophages and monocytes (27). Imiquimod stimulates signal transmission by the nitric oxide induction of synthesizing macrophages (42). The premature antigen-generating cells like DCs are developed and moved to the draining lymph node by identifying pathogens or their constituents via the TLRs. The established DCs show the antigens; further, they trigger ASTC (antigen-specific T cells) and activate the immunity formation, especially antigens with immunologic memory. The immunity generated will activate TLR (s) and affect the unique DC subset (43). The use of TLR agonists as vaccine adjuvants specifies the prominent method in formulating vaccines with improved protective immunity. Considering Imiquimod’s local adjuvant properties in treating infectious diseases in BALB/c mice, it was investigated as a vaccine adjuvant in the experimental model of cutaneous leishmaniasis. It was reported that subcutaneous use of local Imiquimod on the skin before leishmania’s crude antigen immunization increased the infection challenge prevention compared to the crude antigen immunization; it is related to an enhanced Th1 response in opposition to the vaccine antigen (44). More emphasis should be placed on Imiquimod as a vaccine adjuvant for any pathogen antigen delivered intramuscularly, especially when a Th1 response must be activated for protective immunity (45).

**Table 1:** Antigen entrapment, surface charge, particle size, and polydispersity index (PDI) of different liposomal formulations obtained by DLS apparatus

Formulation	Size (nm)	Zeta potential (mv)	PDI	Entrapment efficiency
Empty liposome	217±15	23.5±2	0.238±0.01	-
Liposome+SA	217±15	4.5±3	0.326±0.01	57±14%
Liposome+SA+Imiquimod	217±15	4.5±3	0.365±0.01	57±14%

The mean ± SD is shown in the results (n = 3).

Lane 1: Soluble antigens (SA), Lane 2: Low molecular weight calibration protein (Sigma, USA), Lane 3: Empty liposome, and Lane 4: liposome + SA

On days 42 and 63 following the start of the inoculation, mice were given blood samples. The values are shown as mean ± SD. * denotes a notable distinction between the groupings; * *P*<0.05; ** *P*<0.01; *** *P*<0.001 are all examples.

The ELISA technique was used to measure the amount of IFN-γ and IL-4 released in the culture supernatants after mononuclear splenocytes were cultivated with SA (10 μg/ml). The mean ± SD is displayed for each of the three results. Significant differences between the groups are indicated by the following: * *P*<0.05, ** *P*<0.01, and **** *P*<0.0001.

A *T. gondii* RH strain was used to challenge ten mice per group inoculated with SA, liposome DOTAP + SA, liposome DOTAP + Imiquimod + SA, SA + Imiquimod, or buffer alone. According to the statistics showing mean ± SEM, there were significant differences between the five groups (** *P*<0.01).

## Conclusion

The results revealed that cationic liposomes carrying soluble Toxoplasmosis parasite antigens and Imiquimod adjuvants demonstrated the highest level of cellular immunity. Immune modeling by cationic liposomes could be efficient in enhancing immunization against *T. gondii*, which indicates that to design future vaccines for Toxoplasma, immunostimulatory adjuvants, as well as the impacts of lipid compositions, need to be regarded concerning the immune response types. The authors recommend the cationic liposomes as protective elements and adjuvant of Imiquimod as effective immunomodulators for further studies regarding vaccine design against *T. gondii* and other intracellular parasites.
